# Oral frailty among dentate and edentate older adults in long-term care

**DOI:** 10.1186/s12877-023-04605-7

**Published:** 2024-01-11

**Authors:** Lina Julkunen, Riitta Saarela, Hanna-Maria Roitto, Hannu Kautiainen, Kaisu Pitkälä, Päivi Mäntylä, Kaija Hiltunen

**Affiliations:** 1 Department of Oral and Maxillofacial Diseases, University of Helsinki, Helsinki, Finland; 2 Social Services, Health Care and Rescue Services Division, Oral Health Care, City of Helsinki, Finland; 3 Faculty of Medicine (Clinicum), University of Helsinki, Helsinki, Finland; 4 Geriatric Clinic, Helsinki Hospital, Helsinki, Finland; 5 Population Health Unit, Finnish Institute for Health and Welfare, Helsinki, Finland; 6 Primary Health Care Unit, Kuopio University Hospital, Kuopio, Finland; 7 Folkhälsan Research Center, Helsinki, Finland; 8 Department of General Practice, University of Helsinki, Helsinki, Finland; 9 Unit of Primary Health Care, Helsinki University Hospital, Helsinki, Finland; 10 Institute of Dentistry, University of Eastern Finland, Kuopio, Finland; 11 Oral and Maxillofacial Diseases, Kuopio University Hospital, Kuopio, Finland

**Keywords:** Oral frailty, Oral disease burden, Edentulism, Oral health, Older adults, Long-term care

## Abstract

**Background:**

The objectives of this study were to compare oral frailty (OFr) among edentate and dentate older adults living in long-term care facilities (LTCF) and to clarify how edentulism and oral disease burden (ODB) of dentate older adults are associated with OFr.

**Methods:**

The population of this study comprised 94 edentate and 209 dentate residents in LTCF in Helsinki, Finland, who had previously participated in a nutritional study. The participants underwent a clinical oral examination. The dentate residents were further divided into three ODB groups based on asymptotic dental score. The edentate and different ODB groups were compared with each other regarding demographics and oral and medical findings. OFr was defined as ≥ 2 of following: having a diet of soft/pureed food, residue of food in the oral cavity, inability to keep the mouth open during examination, unclearness of speech, dry mouth. The association between OFr and edentulousness and various levels of ODB was analyzed by a multivariate logistic model.

**Results:**

Participants with low ODB had significantly less OFr than their edentate peers (*p* = 0.009). Furthermore, the edentate and dentate with high ODB had similar odds for OFr.

**Conclusions:**

Edentulousness and high ODB are equally harmful conditions and may predispose to OFr. This study suggests that maintaining healthy natural teeth and good oral health (low ODB) may protect against OFr.

**Trial registration:**

The Ethics Committee of the Hospital District of Helsinki and Uusimaa approved the protocols for the nutritional status and oral healthcare studies and the merging of the data, including patient medical records (Register number HUS/968/2017).

## Background

A healthy mouth is an important part of well-being, comprising several oral functions such as eating, swallowing, speaking, and smiling [1]. Increasingly more adults are reaching advanced age with natural teeth, at least partly due to constant developments in oral healthcare. Although edentulism is declining, many older adults are still edentulous, especially in long-term care facilities (LTCF) [2]. Edentulism and partial tooth loss affect nutrition, quality of life, and well-being and increase the risk for mortality [2–4].

Natural teeth require constant care throughout life. Particularly older adults living in LTCF are often frail, functionally impaired, have dementia, and are dependent on caregivers’ help [5]. Unfortunately, their oral hygiene is often poor due to the lack of knowledge and time of personnel, and hence, oral care is often difficult to implement or neglected [6]. Previous studies show that a large proportion of the residents in LTCF suffer from oral diseases and oral disease burden [7–9]. Deficiency in oral care directly increases the risk of poor oral health and oral diseases such as dental caries, periodontitis, and associated systemic infections [7–9]. Oral diseases and oral biofilm have associations with systemic health and dementia, especially Alzheimer’s disease [10]. The association is bidirectional because Alzheimer’s disease has been found to increase the risk for poor oral health, while cleaning is challenging due to apraxia and cognitive decline [11, 12]. There is also a relationship between poor oral health and aspiration pneumonia, which may lead to death [13].

Frailty is described as impaired functioning, increased vulnerability, and low stress tolerance. It increases the risk for disability, falls, hospitalization, and death [14, 15]. Aging increases the risk of frailty, but studies show that the risk is higher among those with comorbidities, low socioeconomic status, poor diet, and poor oral health [16, 17]. There are various definitions for oral frailty (OFr) but most of them include weakness/weakening of oral muscles and difficulty in chewing, eating, speaking, and swallowing [18, 19]. Some include the number of teeth [20]. Swallowing difficulties are associated with chewing difficulties due to reduced mastication and often co-occur among residents in institutional care [21]. Effects of oral health on general frailty have been studied [16, 22], but its impact on OFr is a less known topic among older adults in LTCF settings. However, OFr has been found to significantly affect life expectancy [23]. Studies show that poor oral health is associated with OFr, and it may affect malnutrition and health-related quality of life (HRQoL) [24–26]. Tooth loss impairs masticatory function and causes oral hypofunction. It is therefore important to prevent oral diseases that may eventually lead to loss of teeth [5, 18, 25, 27]. Losing teeth is associated with malnutrition and weakening of oral muscles, and edentulousness has been linked to OFr through challenges in oral motor functions [28, 29]. Oral muscles play a crucial role in masticatory function and adequate food fragmenting and swallowing. Problems in eating may have a negative effect on digestion and nutrition, which may lead to malnutrition and weaken the quality of life [30, 31].

Studies show that for older adults living in LTCF oral health is poor and oral disease burden (ODB) is high [9, 32], and thus, it is important to understand the link between poor oral health, its endpoint edentulism, and OFr. The objectives of this study were to compare OFr among edentate and dentate older adults living in LTCF and to clarify whether edentulism and ODB of dentate older adults are associated with OFr. The hypothesis of this study is that both poor oral health and edentulism are associated with OFr.

## Methods

The Ethics Committee of the Hospital District of Helsinki and Uusimaa approved the protocols for the nutritional status and oral healthcare studies and the merging of the data, including patient medical records (Register number HUS/968/2017). The project adhered to the guidelines set forth in the Declaration of Helsinki and the Belmont Accord to ensure the safety of human research subjects. All participants or their proxies in case of moderate-to-severe dementia gave written consent.

This was a cross-sectional study, and the study population consisted of residents in LTCF in Helsinki, Finland, who had previously participated in the nutritional study with clinical examinations (n = 550). Participation was voluntary. Individuals needing prophylactic antibiotics and those refusing the clinical examination or unable to cooperate due to severe cognitive decline were excluded. Some of the participants died before the oral examinations began. The final number of participants in the oral examination was 393, all of whom were included in the FINORAL (Finnish oral health studies in older adults) study. For this current study, the 303 individuals for whom the oral examination was completely performed were included. These participants comprised 94 edentate and 209 dentate older adults. The head nurse of the department filled in a questionnaire regarding study participants’ demographic characteristics (age, sex, education) and diagnoses, and data on medications were obtained from medical records. The Charlson Comorbidity Index (CCI) was assessed and calculated as described. CCI takes into account the number and severity of serious diseases that have impact on patient’s prognosis. The higher the points, the more comorbidities and more severe are the diagnoses [33].

The Barthel Index (BI) [34] was calculated to measure performance in activities of daily living (range 0–100). Fried’s phenotype criteria for frailty (FF) were evaluated regarding unintentional weight loss, exhaustion, low physical activity, slowness, and physical weakness. Residents meeting three or more criteria were classified into the frailty group [14]. The Mini Mental State Examination (MMSE) [35] was used to measure cognitive impairment. Of the maximum score of 30 points, 24 points or more indicate normal cognition, whereas 19–23 points indicate mild, 10–18 points moderate, and ≤ 9 points severe cognitive impairment. Residents’ nutritional status was assessed with Mini Nutritional Assessment (MNA) [36] and categorized as good nutrition (24–30 points), being at risk of malnutrition (17–23.5 points), or being malnourished (< 17 points).

Two qualified and calibrated dentists conducted the clinical examination. The dentists were equipped with loupes (Merident Optergo MO Ultralight Flip-up), an attached headlamp (Merident Optergo DeLight LED), and a standard set of sterile instruments. Participants were lying on the bed or sitting in a chair during the examination. The oral examination included visual examination of the oral mucosa, food residues on oral surfaces, calculation of the number of teeth, residual roots, and open caries lesions and root caries visible to the naked eye. Also, the Plaque Index (PI) according to the modified Silness & Loe index (0–4, from 0 = no visible plaque to 4 = whole tooth covered with plaque) and the Gingival Index (GI 0–3, 0 = no inflammation to 3 = severe gingival inflammation) were registered [37]. The periodontal pocket probing depth (PPD) measurements were registered as the deepest PPD for each tooth (< 4 mm, 4–5 mm, ≥ 6 mm), and bleeding on probing (BOP) as yes/no for each tooth. Use of removable dentures, their condition, and need for repair were reported. Salivation was clinically evaluated by signs of oral dryness (modified from Osailan et al.): normal salivation, reduced salivation (mirror sticks to buccal mucosa or tongue, frothy saliva), or dry mouth (glossy appearance of oral palate, lobulated/fissured tongue) [38].

Oral disease burden (ODB) was assessed for all dentate participants. The total number of dentate participants (a person with at least one tooth or visible root remnant in the oral cavity) with all needed findings for asymptotic dental score (ADS) calculation was 209. ADS sums up oral pathologies, and was originally described by Janket et al. [39] to evaluate the association between prevalent coronary heart disease and oral pathologies among dentate individuals. Earlier, we have described earlier in detail how ADS was modified to comprise clinical oral examination variables in the study by Julkunen et al. [9], to include the following in the same study population: (1) dental caries (0 = no caries, 1 = 1–3 caries lesions, 2 = 4–7 caries lesions or one edentulous jaw, 3 = ≥ 8 caries lesions); (2) gingivitis (GI ≥ 1 and/or BOP ≥ 20%; values 0 no, 1 yes); (3) root remnants (values 0 = no root remnants, 1 = one root remnant, 2 = two or more root remnants); (4) number of teeth with deepened periodontal pockets [number of teeth with PPD 4–5 mm plus weighted (multiplied by two) number of teeth with PPD ≥ 6 mm] (values: 0 = no pockets, 1 = 1–3 pocket, 2 = 4–10 pockets, 3 = ≥ 11 pockets). The first three variables were in line with Janket et al. [39]. For the fourth variable, Janket et al. measured a proxy for periodontal disease (yes/no) by using the community periodontal index of treatment need (CPITN) (if at least 2 sextants were recorded as having CPITN ≥ 3 signifying that sextant had periodontal pocket depth ≥ 3.5 mm). No X-rays were taken, and thus, no radiologic findings were included in the scoring. The final ADS score of each dentate participant varied from 0 to 9. Dentate participants were further divided into three ODB groups based on received ADS points: 0–2 was deemed low ODB (group I) (n = 39), 3–4 moderate ODB (group II) (n = 96), 5–9 high ODB (group III) (n = 74). The fourth study group consisted of edentate residents (n = 94). ADS was not calculated for edentate participants because ADS score describes tooth-related oral diseases.

Signs of OFr were a diet of soft or pureed food, residue of food in the oral cavity or denture (on the surface of teeth, on the surface of oral mucosa, or on the surface or under removable dentures), inability to keep the mouth open (opens when persuaded but then closes during the examination, opposes or refuses the examination in its entirety), unclearness of speech (not understandable, does not speak), and dry mouth (the mirror sticks to the buccal mucosa or the tongue, frothy saliva, glassy appearance of the oral palate lobulated/fissured tongue). Each sign of OFr was dichotomized as yes/no. The level of OFr was determined by the number of signs (0–1 sign = no or mild, 2–4 signs = moderate, 5 signs = severe OFr) [19]. We have previously shown that this definition of OFr is related to Fried’s frailty phenotype, general health, nutrition, and need for help with daily activities [19].

Data were presented as means with standard deviation (SD) or as counts with percentages. Statistical comparisons between the dentate and edentate were made by using Chi-square test, t-test, or Mann-Whitney test. Statistical comparisons between edentate and dentate ODB groups were conducted by using analysis of variance (ANOVA) or Chi-square test; Hochberg’s multiple comparison procedure was used to correct significance levels for post hoc testing. The relationship between OFr and ADS was analyzed using logistic regression models. Analyses were adjusted with age and gender. In case of violation of assumptions (e.g. non-normality) for continuous variables, a bootstrap-type method or Monte Carlo *p*-values (small number of observations) for categorical variables were used. Normal distributions were evaluated graphically and with the Shapiro-Wilk W test. In all analyses, statistical significance was set at p ˂ 0.05. The Stata 17.0, StataCorp LP (College Station, TX, USA) statistical package was used for the analysis.

## Results

The mean age of participants was 83 and 73% of them were females. Of participants, 73% had dementia and 53% had oral frailty. Firstly, the study population was divided into two groups, edentate and dentate (see Table 1). The mean age was 84 years (SD 7) for edentate and 83 years (SD 9) for dentate participants. Most participants were women: 76% of edentate and 72% of dentate. The majority of the edentate had less than 8 years of education (70%), while for the dentate the corresponding figure was 38% (*p* < 0.001). Both groups had a mean of 9.0 (SD 3.7) regular medications.

**Table 1:** Demographic and general findings of edentate and dentate participants

	Edentate n = 94	Dentate n = 209	*p*-value
**Demographics**			
Age, years, mean (SD)	84(7)	83(9)	0.09
Females, n (%)	71(76)	150(72)	0.50
Education < 8 years, n (%)	59(70)	73(38)	**< 0.001**
**Health status and functional status**			
Charlson comorbidity index, mean (SD)	2.2(1.1)	2.0(1.3)	0.29
Number of regular medications, mean (SD)	9.0(3.7)	9.0(3.7)	0.98
Fried’s frailty, n (%)	42(45)	97(46)	0.78
Barthel Index, mean (SD)	30.5(25.4)	31.1(26.2)	0.88
Self-performed oral hygiene, n (%)	12(15)	44(22)	0.18
Dementia n (%)	77(82)	143(68)	**0.02**
MMSE*, mean (SD)	12.9(7.3)	14.4(7.4)	0.14
**Findings in oral examination**			
Oral mucosa healthy, n (%)	73(83)	162(79)	0.48
Removable dentures^†^, n (%)	46(57)	40(29)	**< 0.001**
Denture in need of repair, n (%)	21(40)	16(41)	0.89
Oral frailty, n (%)	57(61)	103(49)	0.07
**Nutritional status**			
MNA^‡^, n (%)			0.28
Well-nourished (25–30)	21(25)	37(19)	
Risk of malnutrition (17–24)	54(64)	128(67)	
Malnutrition (< 17)	9(11)	25(13)	

^*^ Mini Mental State Examination

^†^ Use of complete or partial removable dentures

^‡^ Mini Nutritional Assessment

Over half of the edentate had removable dentures (57%), whereas the corresponding figure for dentate participants was 29%. In both groups, 40–41% of the removable dentures were in need of repair. In addition, there was a trend for OFr to be more common among the edentate (61%) than the dentate (49%) (*p* = 0.067). Of the edentate, 82% had dementia, whereas of the dentate 68% were diagnosed as having dementia (*p* = 0.015).

Secondly, dentate participants were further divided into three groups, low, moderate, and high ODB, and compared with edentate participants (Table 2). There was a significant difference between all four groups in education (*p* < 0.001), and use of removable denture (*p* < 0.001). Furthermore, the low ODB group differed significantly from edentate group in having healthier oral mucosa (*p* = 0.004) and suffering less often OFr (*p* = 0.009).

**Table 2:** Findings in edentate and dentate participants divided into different oral disease burden (ODB) groups (low = ADS^*^ 0–2, moderate = ADS 3–4, high = ADS 5–9)

	Edentate (E) n = 94	Dentate n = 209			*p*-value
	Reference	I = low ODB n = 39	II = moderate ODB n = 96	III = high ODB n = 74	multiple comparison [post hoc]
**Demographics**					
Age, years, mean (SD)	84(7)	81(9)	82(8)	84(9)	0.14
Females, n (%)	71(76)	29(74)	62(65)	59(80)	0.13
Education < 8 years, n (%)	59(70)	16(42)	34(39)	23(34)	**< 0.001 [E/I, E/II, E/III]**
**Health status and functional status**					
Charlson comorbidity index, mean (SD)	2.2(1.1)	2.0(1.5)	1.9(1.2)	2.1(1.3)	0.65
Number of regular medications, mean (SD)	9.0(3.7)	9.0(3.4)	9.4(3.8)	8.6(3.7)	0.59
Fried’s frailty, n (%)	42(45)	18(46)	37(39)	42(57)	0.13
Barthel Index, mean (SD)	30.5(25.4)	38.1(28.6)	30.8(25.7)	27.9(25.3)	0.36
Oral hygiene by the participant, n (%)	12(15)	10(26)	21(23)	13(18)	0.39
Dementia, n (%)	77(82)	24(62)	65(68)	54(73)	0.05
MMSE^*^, mean (SD)	12.9(7.3)	16.5(7.8)	14.1(7.6)	13.6(6.8)	0.11
**Findings of oral examination**					
ADS^†^, mean (SD)	n/a^‡^	1.5 (0.6)	3.6 (0.5)	6.0 (1.2)	
Oral mucosa healthy, n (%)	73(83)	38(97)	75(80)	49(69)	**0.004 [E/I]**
Removable dentures^§^, n (%)	46(57)	8(31)	18(29)	14(28)	**< 0.001 [E/I, E/II, E/III]**
Denture in need of repair, n (%)	21(40)	3(38)	9(45)	4(36)	0.96
Oral frailty, n (%)	57(61)	13(33)	45(47)	45(61)	**0.009 [E/I]**
**Nutritional status**					
MNA^||^, n (%)					0.42
Well-nourished (25–30)	21(25)	11(29)	17(19)	9(15)	
Risk of malnutrition (17–24)	54(64)	22(58)	61(68)	45(73)	
Malnutrition (< 17)	9(11)	5(13)	12(13)	8(13)	

^*^ Mini Mental State Examination

^†^Asymptotic Dental Score

^‡^Not available

^§^Use of complete or partial removable dentures

^||^Mini Nutritional Assessment

*P*-values (at significance level 0.05) for comparisons with a reference category were adjusted for multiplicity using Hochberg’s multiple comparison procedure

A significant difference was present in dementia between the edentate (82%) and all dentate participants (68%) (*p* = 0.015; Table 1), but when comparing the different ODB groups with the edentate group the difference did not reach significance (Table 2). In nutritional status defined by MNA, no significant difference was observed between the edentate and dentate. The highest proportion of participants in all groups were at risk of malnutrition (58–73%).

**Fig. 1:**
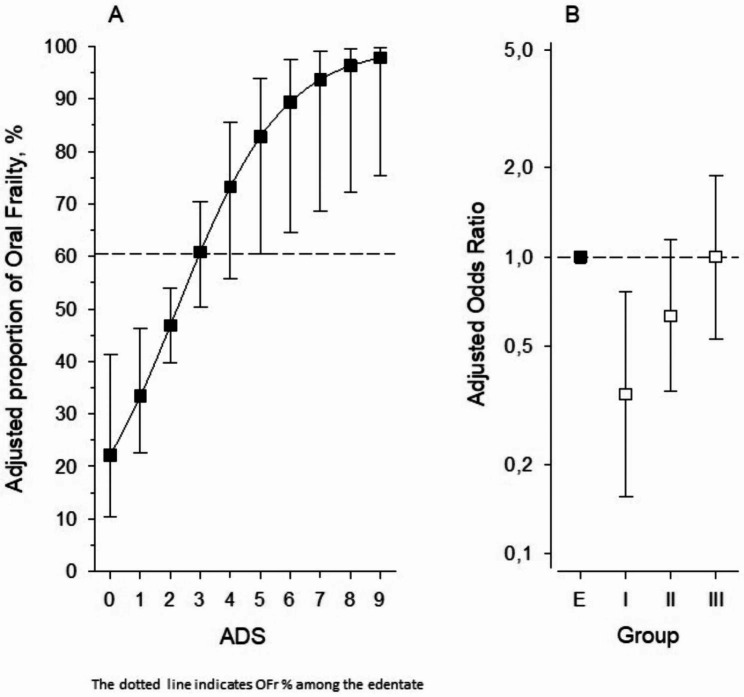
(**A**) Adjusted relationship between oral frailty (OFr) and asymptotic dental score (ADS) as continuous variable (values 0–9) among dentate participants. The dotted line indicates OFr % among the edentate. (**B**) Age- and sex-adjusted odd ratios (ORs) with 95% confidence intervals (CIs) for OFr of dentate participants’ oral disease burden (ODB) groups (I low, II moderate, and III high, defined by ADS) and presented with edentate participants (E) OR 1 as a reference. The estimates were derived from logistic regression models. Whiskers show 95% CIs.

Figure 1A shows the relationship between proportion of OFr and ADS score as a continuous variable (values 0–9) among dentate participants. The proportion of OFr among study participants increased with increasing ADS level. Of the edentate, 61% had OFr, and this incidence was equivalent to ADS level 3 (moderate ODB) among dentate participants. The age- and sex-adjusted 95% confidence interval for odds ratio (OR) for OFr in dentate groups with different levels of ODB is shown in Fig. 1B with edentate group (E) OR 1 as a reference. OR for OFr increased linearly from low ODB (I) to high ODB (III), and the edentates’ reference level was equivalent to the mean OR of high ODB.

## Discussion

The main objective of this study was to analyze OFr between edentate and dentate older adults living in LTCF. Furthermore, the aim was to determine whether edentulism or oral disease burden (ODB) among dentate participants is associated with OFr. Participants were divided according to ADS score into three different ODB groups. The dentate with low ODB had significantly less OFr than the edentate, whereas dentate older adults having high ODB and those who were edentate had similar mean odds for OFr. The hypothesis was that both poor oral health and edentulism are linked to OFr, and the findings of the study support this hypothesis.

Aging is a risk factor for functional impairment and loss of biting force, but it is well known that tooth loss, especially losing posterior teeth, and edentulism may affect biting force [40–42]. In addition to tooth loss, poor oral health weakens oral function, which may cause eating difficulties, malnutrition, and impaired salivary flow [43]. Oral hypofunction and malnutrition have an association with sarcopenia, which may lead to oral dysphagia [44]. OFr means weakness and fatigue of oral muscles, and the definition of OFr in the literature includes such aspects as poor oral health, poor function of oral muscles, risk of weight loss, chewing and swallowing problems, change in food or its composition, difficulties in speaking, and tooth or mouth pain [19, 45]. In our earlier study [19], the signs of OFr were defined as a diet of soft or pureed food, residue of food in the oral cavity or on the denture, inability to keep the mouth open during the oral examination, indication of pain for oral procedures, unclearness of speech, and dry mouth. In the current study, we used five signs of OFr instead of the six listed above [19]. The indication of pain includes the pain caused by periodontal probing of dentate participants, and in this study, it was excluded to avoid the distortion of results between edentate and dentate participants.

The FDI (World Dental Federation) defines oral health as the ability to speak, smile, smell, taste, touch, chew, swallow, and convey a range of emotions through facial expressions without pain, discomfort, or disease of the craniofacial complex [46]. A healthy mouth includes healthy teeth (no active dental caries), healthy periodontium (no gingivitis, no deepened periodontal pockets), good salivary flow, moist and healthy oral mucosa, and sufficient occluding teeth and good biting ability [47]. In this study, the clinically detectable oral disease findings were compiled into a score (ADS). ADS calculation includes all clinically detectable teeth-related disease findings (caries, periodontal diseases, and their final consequences of root remnants and loss of multiple teeth) in a numerically expressed score with values varying from 0 to 9. ADS can be regarded as a surrogate variable of dental status and oral disease burden caused by dentition. In the current study, ADS was useful for the stratification of the dentate study population into three groups with different oral disease burden: no or low, moderate, or high ODB [9]. In our earlier study [9], we showed that all individual variables based on the same stratification of ADS, except edentulous jaw, increased linearly from ODB low to high significantly (*p* < 0.001). In addition, in this earlier study several other oral health-related findings were analyzed, including plaque index, which showed a significant linear increase from ODB low to high (*p* = 0.008) [9].

Previous studies indicate that poor oral health is associated with general frailty [14–16, 18, 19, 22]. However, the association between OFr and oral health has not been extensively investigated. In our study, participants with low ODB had the least OFr, with OFr increasing linearly between ODB groups. In addition, in the pairwise comparisons of dentate groups with the edentate group, the occurrence of OFr was at a similar level for edentate and high ODB groups. This result was further supported by sex- and age-adjusted logistic regression analysis, according to which the odds ratio of OFr for the edentate was equivalent to the mean odds ratio of the dentate with high ODB (Fig. 1B). Based on this, we conclude that having high ODB and being edentate weaken oral function and that good oral health (low ODB) likely protects against OFr.

We defined general frailty with Fried’s frailty phenotype assessment components [14]. An association between frailty phenotype and OFr has been found previously [19]. In this study, Fried’s frailty did not reveal a significant difference between edentate and dentate groups in any comparisons, unlike OFr. Those dentate with the poorest oral health and those who had already lost their natural teeth had a similar risk of OFr and the removable dentures were commonly in need of repair or were not in use at all. Thus, according to our findings, general weakness and OFr may also act as separate phenomena even though they are associated according to a previous study [19]. That can be an important observation to prevent and detect oral and general frailty [24, 29, 44, 45].

While we have no documentation about study participants’ earlier oral disease events, we can assume that the edentate subjects have previously had oral diseases that caused ODB. Earlier studies have shown that the most common reasons for tooth loss are dental caries, apical periodontitis, and periodontitis [48, 49]. Especially periodontitis is strongly associated with systemic diseases [8]. Those who are currently edentate have been exposed to varying degrees of ODB with oral local and systemic effects of infection and inflammation before tooth extractions, which can be a background factor in OFr even though edentate individuals might currently be free of oral inflammation. Edentate participants were significantly lower educated than the dentate groups in this study, consistent with a previous study that found social factors to be more clearly associated with edentulism than factors related to general health [50].

The residents in LTCF often have impaired cognition, and dementia is common. In previous studies, edentulism was associated with low cognition, and Alzheimer’s disease was linked to higher oral bacterial load and inflammation levels [51]. Alzheimer’s disease increases the risk for tooth loss, as oral cleaning is neglected or even forgotten when functional capacity is impaired. In a pairwise comparison of the dentate and edentate, the former significantly less often suffered from dementia than the latter, but in comparisons between various dentate groups and edentate the statistical significance was not so clear. In our study population with high dementia prevalence (82% of the edentate, 62–73% of the dentate in linear order from low to high ODB) and at this end-stage of life, the role of dementia in OFr may be challenging to distinguish. We found no significant difference in MNA, MMSE, or sex in dentate/edentate residents.

A strength of this study is that the data comprise a fairly large number of older adults in long-term care whose oral health status was comprehensively examined by dentists. Furthermore, there was a separate group of edentate residents in our data. We used a score that compiles the clinically detectable oral disease findings (ADS), with which we determined the ODB of the dentate participants [9]. The main limitation of the study is that it is cross-sectional, and we do not have information on participants before their admission to LTCF. As X-rays could not be taken, it is probable that some oral infections, such as periapical lesions, went undetected. In addition, there were relatively few participants with low ODB. Thus, the power of the study may be too low to detect differences between some clinically important variables.

## Conclusion

This study suggests that maintaining natural teeth with good oral health (low ODB) may protect against OFr. Edentulousness and high ODB can equally predispose to OFr. Maintenance of healthy natural teeth and good oral health throughout life and prevention of oral diseases and tooth loss may protect the individual from OFr.

## Data Availability

Data may be available from the authors upon reasonable request and with permission of the City of Helsinki. Authors to be contacted are Lina Julkunen, Kaija Hiltunen and Päivi Mäntylä.

## Abbreviations

ADS: Asymptotic Dental Score
BOP: Bleeding on probing
FINORAL: Finnish oral health studies in older adults
GI: Gingival Index
LTCF: long-term care facility
MMSE: Mini Mental State Examination
MNA: Mini Nutritional Assessment
ODB: oral disease burden
OFr: oral frailty
PI: Plaque Index
PPD: Pocket probing depth
SD: Standard deviation
